# Synthesis, spectroscopic, thermal, crystal structure properties and characterization of new Hofmann-type-like clathrates with 4-aminopyridine and water

**DOI:** 10.3906/kim-2011-29

**Published:** 2021-06-30

**Authors:** Zeki KARTAL, Onur ŞAHİN

**Affiliations:** 1 Retired Professor of Atomic and Molecular Physics, Kütahya Turkey; 2 Department of Occupational Health and Safety, Faculty of Health Sciences, Sinop University, Sinop Turkey

**Keywords:** Hofmann-type clathrates, Hofmann-type-like clathrates, 4-aminopyridine, vibration spectra, single crystal X-Ray diffraction (SC-XRD) analysis

## Abstract

In this study, synthesis of two new heteronuclear tetracyanonickelate(II) clathrates based on 4-aminopyridine (4AP) and guest water (H_2_O) molecule and investigation of their structural properties were reported. These clathrates were characterized by using vibration spectroscopy, elemental, thermal analysis and single crystal X-ray diffraction (SC-XRD) techniques. Examining the elemental and spectral data of these clathrates, it was observed that the formulas [Zn(II)(4AP)_2_Ni(µ-CN)_2_(CN)_2_]·6H_2_O and [Cu(II)(4AP)_4_Ni(µ-CN)_2_(CN)_2_]·H_2_O were defined their structures. General information about the structural properties of these clathrates in single crystal form has been obtained by considering the changes in the characteristic peaks of the cyanide group and the 4AP that formed them. The thermal behaviors of these clathrates were obtained by examining the temperature-dependent changes of their masses. The magnetic susceptibilities of these clathrates in single crystal form were measured with a Gouy balance. According to the data obtained using SC-XRD technique, the heterometallic [Zn(II)(4AP)_2_Ni(µ-CN)_2_(CN)_2_]·6H_2_O compound has *Cmcm* and the heterometallic [Cu(II)(4AP)_4_Ni(µ-CN)_2_(CN)_2_]·H_2_O compound has crystal structures in the *C2* / *c* space group.

## 1. Introduction

Young chemist Karl Andreas Hofmann (1870–1940) first synthesized an interesting compound in 1897 during his studies [1]. This interesting compound had a two-component structure and was called “clathrate” in science. One of the components that make up a clathrate is a “host structure” and the other is an appropriately sized “guest molecule” that enters this host structure [2,3].

The host structure which is one of the components that make up a clathrate can be named in different ways depending on the type of this host structure and the ligand molecule forming the clathrate, e.g., Hofmann-type complex, Hofmann-T_d_-type complex, Werner-type complex, etc.

As a result, the number of clathrates formed by a particular ligand molecule and metal atom is determined by the number of different guest molecules present in the formed host structure. Today, in the scientific field, there are many clathrates given with many different names and many host structures named after these clathrates. As new scientific studies continue on this subject, many new host structures and new clathrate types are still being discovered.

The general formula of Hofmann-type complexes obtained in many important organic and inorganic studies that are of interest to chemistry science is given as M(II)LMʹ(II)(CN)_4_. Here, the letters M and Mʹ denote transition metal (TM) atoms of +2 valence, and the letter L denotes one bidentate or two monodentate ligand molecules. Hofmann-type complexes form 1D, 2D and even 3D polymeric layers according to the bonds made between [Mʹ(CN)_4_]^2–^ anions and [M(L)]^2+^ cations [4].

If a guest molecule enters the cavities of a Hofmann-type complex, then the new structure that is formed is called the “Hofmann-type clathrate”. The general formula of Hofmann-type clathrates is given as M(II)LMʹ(II)(CN)_4_.nG depending on the formula of the Hofmann-type complex. In this formula, G indicates a guest molecule that enters the Hofmann-type host structure and “n” indicates the number of guest molecule in the clathrate [2–4].

The examples of the applications of Hofmann-type complexes and Hofmann-type clathrates in many different fields of science are increasing day by day. Some of the usage areas of Hofmann-type complexes and clathrates; storage of various poisonous substances, gases and radioactive elements in a way to protect the environment, making molecular sieves to separate molecules of the desired size from mixtures of various molecules of different sizes, desalination of sea water to obtain drinking water, to produce of new battery types that are more useful than conventional batteries in terms of size and capacity, to make more sensitive chemical sensors, to obtain and store simpler and cheaper hydrogen gas to protect the nature from pollution and meet its future energy needs, to obtain stronger magnetic materials for storage of larger amounts of energy in future smaller volumes, to obtain new compounds that show superconductivity at normal temperatures to reduce losses in power lines during energy transmission, etc.

During the formation of Hofmann-type complexes and Hofmann-type clathrates, many different interactions occur between the various components that make up them. These interactions are the reason for a number of important features of the Hofmann-type complexes and the Hofmann-type clathrates and these important features are examined with various spectroscopic techniques. Vibration (infrared and Raman) spectroscopy is one of the leading experimental methods that can reveal the properties and host-guest interactions of Hofmann-type complexes and Hofmann-type clathrates [5].

In our previous studies, we obtained in powder and crystal forms some new Hofmann-type complexes using 3-aminopyridine as the ligand molecule. The results obtained regarding these studies have been published in some scientific journals [6,7].

The aim of this study is to create new Hofmann-type-like complexes and Hofmann-type-like clathrates in crystal structure by using 4-aminopyridine as ligand molecule, zinc and copper (TM) atoms and [Ni(CN)_4_]^2−^anion.

A chemical substance with an amino group attached at the para position to the nitrogen atom in a pyridine ring and having the closed chemical formula (C_5_H_6_N_2_) is called 4-aminopyridine (4AP). Other most widely known aminopyridine compounds are 2-aminopyridine (2AP) and 3-aminopyridine (3AP). All aminopyridines are widely used in the treatment of various diseases and for obtaining many new chemical compounds. Some sources about pyridines and aminopyridines derived from them, and some previous scientific studies on them are listed in the references section [6–29].

When the periodic table is examined, it is seen that copper and zinc elements are present in the 4th period. This period is the initial period of a very important group of elements known as TMs. The copper is a metal with very high thermal and electrical conductivity. The copper is essential to all living organisms as it is an essential component for vital activities [30].

In terms of human health, metal of zinc is not as toxic as some other metals. Metal of zinc is an important trace element for humans as well as for all other organisms. Sufficient metal of zinc is absolutely necessary for cell growth and division in all organisms, that is, for the continuation of life [30].

Generally, cyanonickelate compounds are a group of anions composed of nickel atoms and cyanide ligands. The most important of the cyanonickelate compounds are tetracyanonickelates, which have four cyanide groups per nickel atom. The tetracyanonickelates have a square planar geometry formed by dsp^2^ hybridization and contain the diamagnetic [Ni(CN)_4_]^2−^ anion [31].

In literature studies, the Hofmann-type complexes and different kinds of complexes obtained by other researchers using 4AP were encountered. However, the vast majority of the complexes obtained by other researchers are in powder form, and those obtained in the form of a single crystal are very few [13–29].

In this study, 4AP, TM salts zinc(II) acetate dehydrate [Zn(OOCCH_3_)_2_·2H_2_O] and copper(II) acetate monohydrate [Cu(OOCCH_3_)_2_·H_2_O] and potassium tetracyanonickelate monohydrate [K_2_Ni(CN)_4_·H_2_O] compounds were used to obtain new Hofmann-type clathrates whose structures conform to the formula M(4AP)_2_Ni(CN)_4_·nH_2_O. As a result of our works, two new Hofmann-type-like clathrates were obtained in crystalline form, which chemical formulas were considered to be [Zn(II)(4AP)_2_Ni(CN)_4_]·nH_2_O and [Cu(II)(4AP)_2_Ni(CN)_4_]·nH_2_O.

## 2. Experimental

### 2.1. Materials

The chemicals used in this study can be listed as 4AP (Sigma-Aldrich, St. Louis, MO, USA; 99%), Zn(OOCCH_3_)_2_·2H_2_O, (Sigma-Aldrich; 99%), Cu(OOCCH_3_)_2_·H_2_O, (Sigma-Aldrich; 99+%), K_2_[Ni(CN)_4_]·H_2_O, (Fluka, Buchs,Switzerland; 96%) and ammonia solution (Merck, Darmstadt, Germany; NH_3_, 25%). All these chemicals were taken from different sources and used without any additional process.

### 2.2. Syntheses of Hofmann-type-like clathrates [Zn(II)(4AP)2Ni(CN)4]·nH2O and [Cu(II)(4AP)2Ni(CN)4]·nH2O

First, 1 mmol of K_2_[Ni(CN)_4_]·H_2_O (0.259 g) was dissolved in distilled hot water (10 mL) and 2 mmol of 4AP (0.188 g) was added to this solution. Then, a solution of 1 mmol M(II)(OOCCH_3_)_2_ [M(II)(OOCCH_3_)_2_ = Zn(OOCCH_3_)_2_·2H_2_O (0.220) g or Cu(OOCCH_3_)_2_·H_2_O (0.200 g)] in distilled hot water (5 mL) was added to the mixture. Water used as a solvent in the formation of Hofmann-type complexes is always present in large amounts in the reaction medium. Due to the fact that the structures of the water molecules are small enough and are abundant in the environment, the water molecules can enter the cavities of Hofmann-type complexes as guest molecules. As a result of all these chemical reactions, Hofmann-type-like clathrates, which are thought to be their formulas as [Zn(II)(4AP)_2_Ni(CN)_4_]·nH_2_O and [Cu(II)(4AP)_2_Ni(CN)_4_]·nH_2_O were formed in suspension form in aqueous media. The diluted ammonia solution was added to the resulting complexes to obtain cleaner and more transparent mixtures. These transparent and clear mixtures were stirred with magnetic stirrer for 4 h at approximately 65 °C and filtered to remove impurities in them and allowed to crystallize under normal conditions.

As a result of this study, two transparent-looking crystalline compounds, one colorless and the other blue, were obtained approximately one to one and a half months later. These compounds are Hofmann-type-like clathrates whose formulas are thought to be [Zn(II)(4AP)_2_Ni(CN)_4_]·nH_2_O and [Cu(II)(4AP)_2_Ni(CN)_4_]·nH_2_O, respectively.

The complexes formed by the cyanide ligand, the metal(II) atoms and other ligand molecules show high structural variability due to the presence of the cyanide ligand in both terminal and bridge forms in these complexes [5,17].

According to the results of elemental analysis and SC-XRD examinations of the structures of these Hofmann-type-like 4AP water clathrates obtained, it was found that their full formulas were [Zn(II)(4AP)_2_Ni(µ-CN)_2_(CN)_2_]·6H_2_O ( **1** ) and [Cu(II)(4AP)_4_Ni(µ-CN)_2_(CN)_2_]·H_2_O ( **2** ) respectively.

In this study, metal(II) atoms, ligand molecules and [Ni(CN)_4_]^2−^anion ratios were taken as 1:2:1 in order to obtain the Hofmann-type-like host structure of the compounds. It has been observed that this ratio occurs at the same values in complex **1** , but at a different value in complex **2** , i.e. 1:4:1. This result either naturally occurred at the time of the chemical reaction, or this rate was mistakenly taken by us.

**Figure 1:**
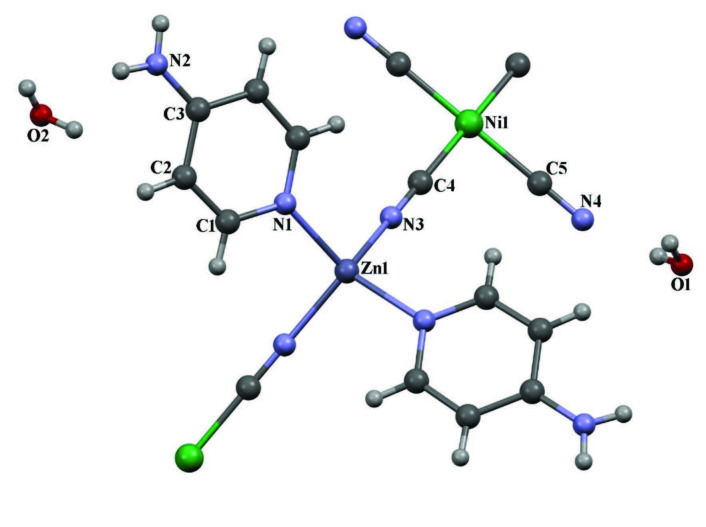
The molecular structure of complex **1** showing the atom numbering scheme.

**Figure 2:**
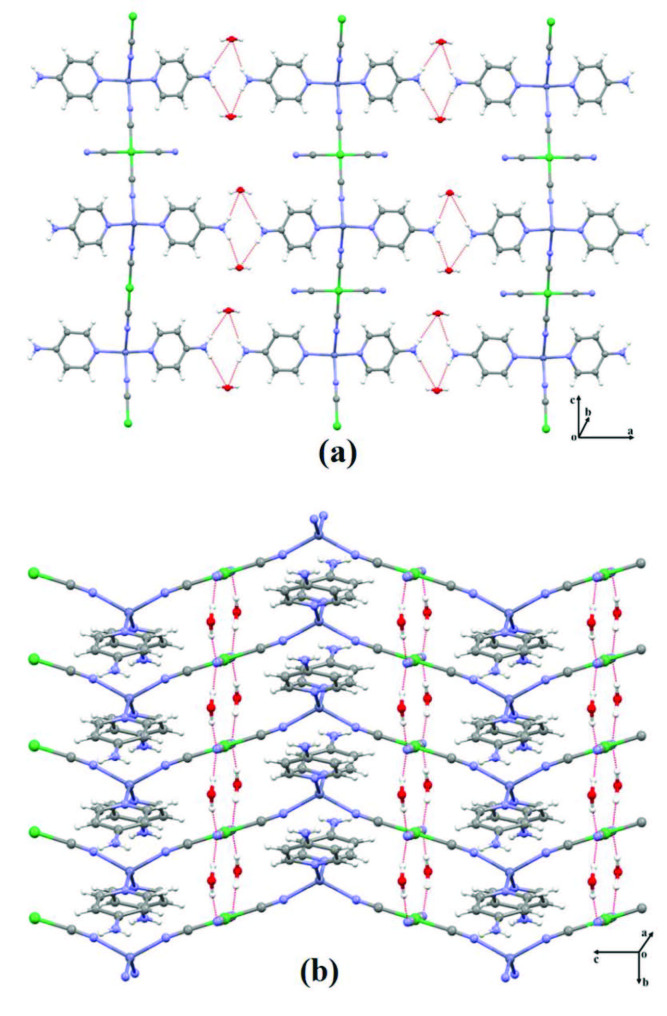
Views of infinite 2D network in complex **1** from ac plane (a) and *bc* plane (b).

According to these experimental results, the complex **1** is a fully suitable example for the cyano-bridged heteropolynuclear complexes and Hofmann-type-like water clathrates. Because two of the four cyanide ligands in complex **1** are in the bridge state, while, the other two are in the terminal state (see Figures 1 and 2). The complex **2** is also an example of clathrate resembling Hofmann-type-like water clathrates. Because, as expected in Hofmann-type compounds, the number of 4AP in the structure of complex **2** is not 2, that number is 4. The value of “n”, which indicates the number of guest molecules in clathrates, may take a certain positive value greater than zero depending on various conditions.

### 2.3. Instrumentation

FT-IR spectra of the complexes were obtained with the Bruker Optics Vertex 70 FT-IR spectrometer (Bruker Optics, Ettlingen, Germany) in the wavenumber range of (4000–250) cm^–1^ at 2 cm^–1^ resolution using the KBr technique under normal laboratory conditions. FT-Raman spectrum of the complex **2** was obtained at under normal laboratory conditions with a Bruker Senterra dispersive Raman microscope using the 532-nm line of a 3B diode laser in the wavenumber range of (4000–100) cm^–1^. Despite all efforts, the FT-Raman spectrum of complex **1** could not be obtained. In each attempt, the sample was disrupted by the effect of laser light. The data of the crystal structures of the complexes **1** and **2** were collected with a D8-QUEST diffraction meter equipped with a graphite-monochromatic Mo-K_α _(λ = 0.71073 Å) radiation. The thermal curves of complexes **1** and **2** were recorded in a nitrogen environment at a heating rate of 10 ^o^C/min and in the temperature range (25–850) ^o^C using platinum crucibles on a SETARAM LABSYS evo thermal analyzer (SETARAM Instrumentation, Caluire, France). Magnetic susceptibility measurements of the complexes **1** and **2** were taken at normal laboratory conditions using a Sherwood Scientific Magway MSB MK1 model magnetic balance (Sherwood Scientific, Cambridge, UK) by the Gouy method using Hg[Co(SCN)_4_] as a calibrating agent.

For this study, the metal amounts in the structure of the complexes obtained were analyzed with the Perkin-Elmer optima 4300 DV ICPOES device (PerkinElmer, Waltham, MA, USA) and the carbon, nitrogen and hydrogen amounts were analyzed with the CHNS-932 (LECO Corporation, St. Joseph, MI, USA) elemental measuring device. The results obtained from these measurements are shown in Table 1.

**Table 1:** Elemental analysis of complexes 1 and 2.

The clathrates and molecular weight Mr (g)	Elemental analysis, found (%)/ (calculated) (%)					
	C	H	N	Zn	Cu	Ni
[Zn(II)(4AP)_2_Ni(µ-CN)2(CN)2]·6H2O; Mr = 524.49	31.68(32.06)	4.29(4.61)	20.98(21.37)	11.86(12.47)	-(-)	10.53(11.19)
[Cu(4AP)4Ni(µ-CN)2(CN)2]·H2O; Mr = 620.82	45.52(46.44)	3.94(4.22)	26.85(27.08)	-(-)	10.39(10.24)	9.21(9.46)

## 3. Results and discussion

### 3.1. Crystallographic analyses of complexes 1 and 2

The H atoms of C atoms were treated as riding atoms with distances of 0.93 Å. Other H atoms were refined freely. We used these procedures for our analysis: solved by direct methods; SHELXS-2013 [32]; refined by full-matrix least-squares methods; SHELXL-2013 [33]; data collection: Bruker APEX2 [34]; molecular graphics: MERCURY [35]; solution: WinGX [36]. Details of data collection and crystal structure determination are given in Table 2. 

**Table 2:** Crystal data and structure refinement parameters for complexes 1 and 2.

Crystal data	1	2
Empirical formula	C_14_H_24_N_8_O_6_NiZn	C_24_H_26_N_12_OCuNi
Formula weight	524.49	620.82
Crystal system	Orthorhombic	Monoclinic
Space group	Cmcm	C2/c
a (Å)	27.894 (10)	20.345 (4)
b (Å)	4.0176 (12)	17.357 (4)
c (Å)	18.455 (6)	19.452 (5)
b(º)	90.00	121.508 (13)
V (Å3)	2068.2 (11)	5857.0 (2)
Z	4	8
Dc (g cm–3)	1.684	1.408
θ range (º)	2.9–26.3	3.1–28.5
Measured refls.	12039	69180
Independent refls.	1072	5701
Rint	0.088	0.069
S	1.30	1.34
R1/wR2	0.096/0.248	0.126/0.229
Drmax/Drmin (eÅ–3)	0.68/–1.41	1.26/–1.85

#### 3.1.1. Complex 1

The SC-XRD study shows that heterometallic complex **1** has 1D coordination polymer. The asymmetric unit of the heterometallic complex **1** consists of one Ni(II) ion, one Zn(II) ion, two cyanide ligands, half 4AP and one and half guest water molecules as seen in Figure 1. The complex **1** has *Cmcm* crystal structure in the space group. The Zn(II) ion is located on inversion center and coordinated by two nitrogen atoms [Zn1-N3 = 1.987(10) Å] from cyanide ligands and two nitrogen atoms [Zn1-N1 = 2.010(11) Å] from 4AP, thus showing a distorted tetrahedral coordination geometry. In experimental data for other complex structures these Zn-N and other metal distances between the Zn(II) and Fe(II) metal ion and the nitrogen atom of the cyanide ligands were found by different researchers to be 2.174(3), 2.131(3), 2.15(5) and 2.375(17) Å, respectively [7,11,37]. In theoretical and experimental calculations for other complex structures these Zn-N distances between the Zn(II) metal ion and the nitrogen atom of the 4AP and similar ligand [with Fe(II) atom] were found by different researchers to be 2.0000, 2.0102 and 2.208(7) Å, respectively [15,28,11].

 The Ni(II) ion is coordinated by four carbon atoms [Ni1-C4 = 1.858(13) Å and Ni1-C5 = 1.868(14) Å] from cyanide ligands, thus showing a square planar coordination geometry. These Ni-C distances belonging to the Ni(CN)_4_ group were found to be approximately 1.865(4), 1.877(3) and 1.881(3) Å in complex structures by other researchers with different metal atoms and different ligand molecules [37,38]. As can be clearly seen from Figures 1 and 2, two of the four cyanide groups in the square planar Ni(CN)_4_ structure, mutually positioned with respect to the nickel atom, are attached to the zinc metal atoms by their nitrogen atoms. Because of this bonding, a bridged compound structure is formed. The other two cyanide groups in the square planar Ni(CN)_4_ structure, mutually form two terminal points with respect to the nickel atom. The presence of two different cyanide groups in the same complex structure and connected to the same metal atom in bridge and terminal state causes CN stretching vibration peaks at two different wavenumbers in the vibration spectrum of that complex (see subsubsection 3.2.2).

 The Zn(II) and Ni(II) ions and cyanide ligands are 1D coordination polymer running parallel to the [001] direction as seen in Figure 2. Adjacent 1D coordination polymers are joined by N-H···O hydrogen bonds, generating 2D supramolecular network which is running parallel to the *ac* plane (Figure 2a). Similarly, these 1D coordination polymers are joined by O-H···N hydrogen bonds, generating 2D supramolecular network which is running parallel to the *bc* plane (Figure 2b).

These two different hydrogen bonds, clearly seen in Figure 2, cause the structure of complex **1** to be formed in 3D.

#### 3.1.2. Complex 2

**Figure 3:**
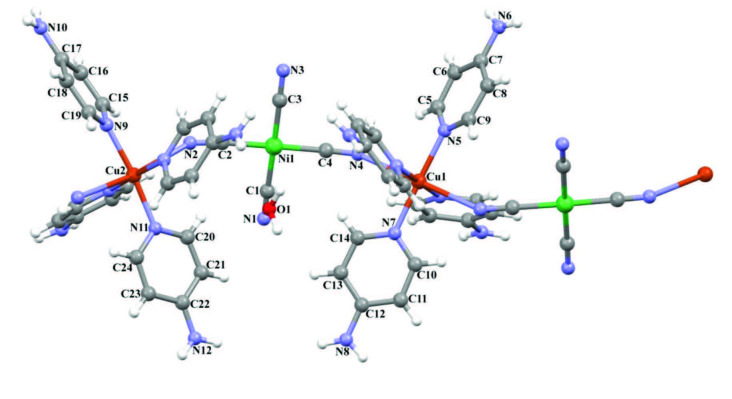
The molecular structure of complex **2** showing the atom numbering scheme.

The asymmetric unit of heterometallic complex **2** contain two Cu(II) ions, one Ni(II) ion, four 4AP, four cyanide ligands and one noncoordinated guest water molecule as seen in Figure 3. The complex **2** has crystal structure in the *C2/c* space group. Each Cu(II) ion is located on inversion center and coordinated by four nitrogen atoms [Cu-N bond range between 2.026(7) and 2.041(8) Å] from 4AP and two nitrogen atoms [Cu1-N2 = 2.692(4) Å and Cu1-N4 = 2.695(4) Å] from cyanide ligands, thus showing a distorted octahedral coordination geometry. These Cu-N distances were found by different researchers to be 2.000(1), 2.010(2) and 2.4625(19)Å, respectively [18,28,39].

The Ni(II) ion is coordinated by four carbon atoms [Ni-C bond range between 1.848(11) and 1.867(8) Å] from cyanide ligands, thus showing a square planar coordination geometry. These Ni-C distances have been found by other researchers to be approximately 1.865(4), 1.877(3) and 1.881(3) Å in various complex structures, just as in complex **1** [37,38].

**Figure 4:**
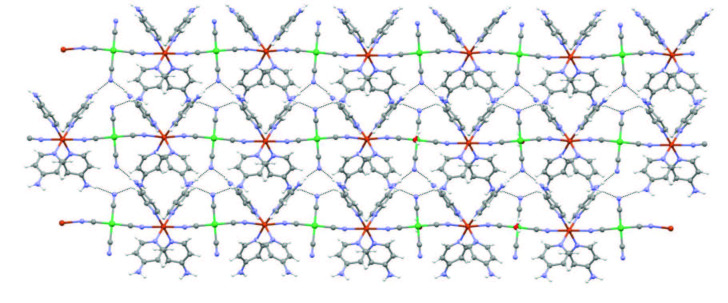
An infinite 2D layer in the complex **2**.

As can be clearly seen from Figures 3 and 4, two of the four cyanide groups positioned with respect to the Ni(II) atom in the square planar Ni(CN)_4_ structure are linked by N atoms to Cu(II) metal atoms. Because of these bonds, a bridged compound structure is formed. The other two cyanide groups mutually form two terminal points relative to the Ni(II) atom. In other words, in this crystal structure, the bonding of both the bridge cyanide group and the terminal cyanide group to the same Ni(II) metal atom causes the CN stretching vibration peaks to split into two.

As can be clearly seen from the Figure 4, while two of the four cyanide ligands in the Ni(CN)_4_ group in the structure of complex **2** are connected to the 1D coordination polymer as bridge groups. Other two of cyanide ligands are positioned as opposed terminal groups at perpendicular angles to this 1D coordination polymer. The nitrogen atoms of the two cyanide ligands positioned as terminal groups are each linked with a hydrogen bond to a hydrogen atom of the NH_2_ groups of the two 4AP in the other 1D coordination polymers.

 The Cu(II) and Ni(II) ions and cyanide ligands are 1D coordination polymer running parallel to the [100] direction. Adjacent these 1D coordination polymers are further joined by N-H···N hydrogen bonds. The combination of these interactions produce 2D network as seen in Figure 4.

The bond distances, bond angles between some selected atoms and hydrogen-bond parameters in the complexes **1** and **2** are given in Tables 3 and 4, respectively.

**Table 3:** Selected bond distances and angles for complexes 1 and 2 (Å, º).

Complex 1			
Ni1-C4	1.858(13)	Ni1-C5	1.868(14)
Zn1-N3	1.987(10)	Zn1-N1	2.010(11)
N3-Zn1-N3i	124.5(7)	N3-Zn1-N1iii	100.77(19)
N3-Zn1-N1	100.8(2)	N1-Zn1-N1iii	132.7(7)
Complex 2			
Cu1-N5	2.026(7)	Cu1-N7	2.040(7)
Cu2-N9	2.029(7)	Cu2-N11	2.041(8)
Cu1-N2	2.692(4)	Cu1-N4	2.695(4)
Ni1-C1	1.855(10)	Ni1-C2	1.863(8)
Ni1-C3	1.848(11)	Ni1-C4	1.867(8)
N5-Cu1-N7	178.0(3)	N9-Cu2-N11	173.7(3)

Symmetry codes: (i) x, y, −z+1/2; (iii) −x+1, y, −z+1/2 for the complex 1.

**Table 4:** Hydrogen-bond parametersand angles (Å

D-H· · ·A	D-H	H···A	D···A	D-H···A
Complex 1				
O1—H1A···N4	0.83 (2)	2.11 (5)	2.913 (15)	163
N2—H2A···O2	0.83	2.19	2.985 (17)	163
Complex 2				
C10—H10···N4i	0.93	2.54	3.198 (12)	128
C24—H24···N2ii	0.93	2.40	3.150 (12)	138
N6—H6B···N1iii	0.86 (2)	2.28 (4)	3.125 (17)	169
N8—H8B···N3iv	0.86 (2)	2.27 (7)	3.049 (15)	150
N10—H10B···N1v	0.86 (2)	2.31 (5)	3.146 (16)	163
N12—H12B···N3iv	0.86 (2)	2.22 (5)	3.047 (14)	163

Symmetry codes: (i) −x+1, y, −z+1/2; (ii) −x+2, y, −z+1/2; (iii) x−1/2, y−1/2, z; (iv) −x+3/2, y+1/2, −z+1/2; (v) x+1/2, y−1/2, z for complex 2.

**Figure 5:**
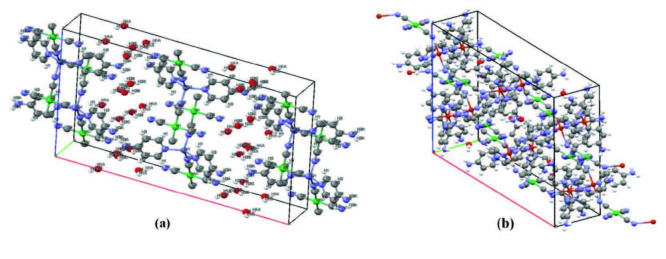
Unit cells of complex **1** (a) and **2** (b).

The unit cells of complexes **1** and **2** are shown in the Figures 5a and 5b, respectively. Atom labels are removed in Figure 5b to provide a more comfortable view.

### 3.2. Spectral characterization of complexes 1 and 2

**Figure 6:**
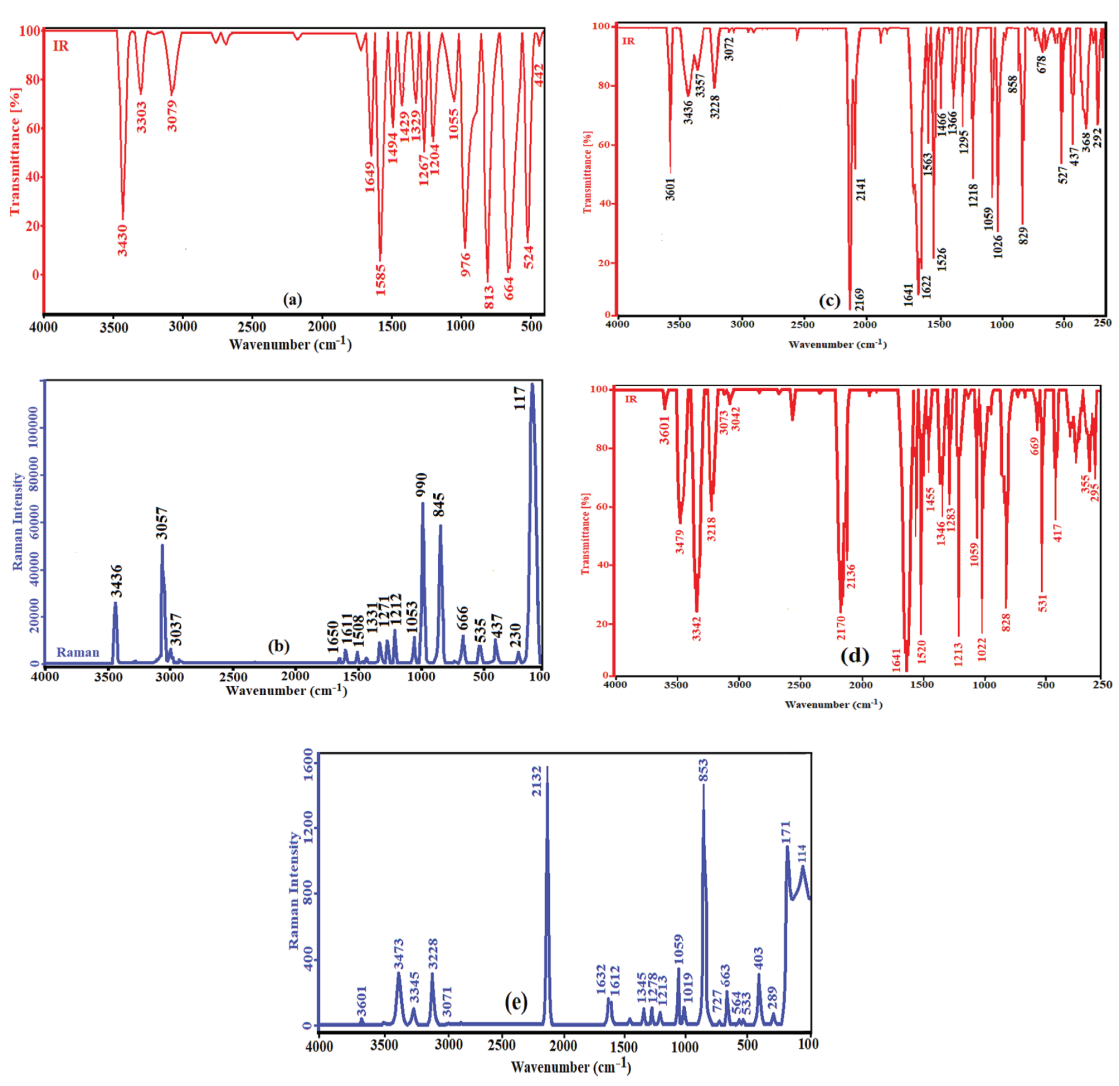
The vibration spectra of the 4AP; FT-IR spectrum (a), FT-Raman spectrum (b), FT-IR spectrum of the complex 1 (c) and the vibration spectra of the complex 2; FT-IR spectrum (d) and FT-Raman spectrum (e).

Vibration spectra of 4AP (Figures 6a and 6b), FT-IR spectrum of complex **1** (Figure 6c) and vibration spectra of complex **2** (Figures 6d and 6e) are given in Figure 6 in this section. We were unable to give the FT-Raman spectrum of complex **1** because in every process performed to obtain the FT-Raman spectrum of complex **1** , the sample of complex **1** was distorted by the effect of the laser light used.

In addition, the similarities of the spectra are immediately striking from the examination of the spectra of the obtained complexes **1** and **2** . The presence of vibration bands of 4AP in the vibration spectra of each of the complexes is the biggest evidence that 4AP are also present in the structures of the complexes **1** and **2** .

Spectral data of the complexes can be analyzed separately for vibrations of the 4AP, [Ni(CN)_4_]^2−^ anions and the guest H_2_O molecule, respectively.

#### 3.2.1. Vibrations of the 4AP

Various studies conducted by many researchers with the 4AP are encountered in the literature [13–29]. Especially Akyüz et al. studied Hofmann-type complexes composed through combination of 4AP with TM atoms [15,18]. These Hofmann-type complexes were generally obtained in powder form in the studies of the relevant researchers. Some other researchers have also explored the crystal structures of a number of complexes consisting of 4AP, some other ligand molecules, and different metal atoms in recent years [11,13,17,28]. Now, in this study, we will examine the complexes **1** and **2** , which are examples of the Hofmann-type-like 4AP water clathrates that we obtained using the 4AP, Zn(II) and Cu(II) metal atoms, [Ni(CN)_4_]^2−^anions, and water molecule as a guest molecule.

The 4AP has a planar structure and belongs to the *C* *_2v_* symmetry. It has 33 normal modes of vibration [16,18,22,29]. The 4AP has unshared electron pairs on the nitrogen of pyridine ring and NH_2 _groups. So the 4AP may be act as a bidentate ligand [14,17]. However, according to structure analysis of the complexes **1** and **2** , it appears that the 4AP binds to the TM atom from the nitrogen atom on the pyridine ring and from the hydrogen atoms of the NH_2_ group to the nitrogen atom of the cyanide ligands [20,21]. 

Some of these 33 normal vibration modes of the 4AP are strongly influenced by its composition with other molecules or metal atoms [16,18,22,26]. Examples of these vibration modes are the stretching and bending vibrations of the NH_2_ group [28,37–39] and the stretching with in plane bending vibrations of the [C(1)═N(1)-C(5)] group which at the pyridine ring [18,25,26]. The wavenumbers (or at the same time frequencies) of these vibration modes shift a considerable amount of either to a high value or to a low value by the combination of the 4AP with other molecules or metal atoms in the complexes **1** and **2** . Once a compound has been formed, there may be small shifts in the vibration frequencies of some functional groups of the ligand molecule, which, those functional groups have not even participated in compound formation, into the high frequency region or the low frequency region. These small shifts are only because the environmental conditions of these functional groups have changed slightly.

The asymmetric stretching vibration of the NH_2_ group in the free 4AP yields a vibration peak at 3430 and 3436 cm^–1^ wavenumbers in its FT-IR and FT-Raman spectra, respectively, while this vibration peak occurs at 3479 and 3473 cm^–1^ wavenumbers in the FT-IR and FT-Raman spectra of complex **2** , respectively. This vibration peak occurred in the FT-IR spectrum of complex **1** at 3436 cm^–1^ wavenumber. Due to the 4AP forming new complexes, this vibration mode has shifted to the higher wavenumber region of 6 and 49 cm^–1^ in the FT-IR spectra of complexes **1** and **2** , respectively. The same vibration mode occurred at 3473 cm^–1^ wavenumber in the FT-Raman spectrum of complex **2** , and, which shifted to the higher wavenumber region of 37 cm^–1^ relative to its value in that free 4AP.

The symmetric stretching vibration of the NH_2_ group in the free 4AP yields a vibration peak at 3303 and 3306 cm^–1^ wavenumbers in its FT-IR and FT-Raman spectra, respectively, while this vibration peak occurs at 3342 and 3344 cm^–1^ wavenumbers in the FT-IR and FT-Raman spectra of complex **2** , respectively. This vibration peak occurred in the FT-IR spectrum of complex **1** at 3357 cm^–1^ wavenumber. Due to the 4AP forming new complexes, this vibration mode has shifted to the higher wavenumber region of 54 and 39 cm^–1^ in the FT-IR spectra of complexes **1** and **2** , respectively. The same vibration mode occurred at 3344 cm^–1^ wavenumber in the FT-Raman spectrum of complex **2** , and, which shifted to the higher wavenumber region of 38 cm^–1^ relative to its value in that free 4AP. In addition, in the FT-IR spectra of free 4AP and complexes **1** and **2** , a third vibration band appears in the region of the stretching vibrations of the NH_2_ group, at a wavenumber of 3228 and 3218 cm^–1^, respectively. This new vibration bands have been defined by some researchers as the overtone of the bending vibration mode of the NH_2_ group at around 1600 cm^–1^[9] and the symmetrical stretching vibration of the NH_2_ group being splitting into two due to the Fermi resonance [20,21].

Since the shifts in the stretching vibrations of the NH_2_ group due to the complex formation of the 4AP are considerably smaller than 150–200 cm^–1^, it is assumed that the 4AP is not bound from the nitrogen atom of the NH_2_ group in the complexes **1** and **2** [15,16,18]. The reality of this situation can be clearly seen from Figures 1 and 3.

As seen in the Table 5, the stretching vibrations of the CH groups in the free 4AP give some vibration peaks between 3079 and 3004 cm^–1 ^wavenumber in the FT-IR and FT-Raman spectra of the complexes **1** and **2** . However, the changes occurring in these C-H stretching vibrations are considerably smaller than the changes observed in the stretching vibrations of the NH_2_ group. These small changes in the wavenumber of these vibration modes can be attributed to the change of its environmental conditions, since the 4AP forms new complexes.

**Table 5:** The some tentative vibration wavenumbers (cm−1) of 4AP in free state and in complexes 1 and 2.

Assignmenta	Free state 4AP		1	2	
	IR	Raman	IR	IR	Raman
νas(NH2)	3430 vs	3436 m	3436 w	3479 m	3473 w
νs(NH2)	3303 w	3306 vw	3357 w	3342 s	3344 m
ν(C-H)	3079 w	3057 m	3072 vw	3073 vw	3071 m
ν(C-H)	-	3037 w	3044 vw	3042 vw	3037 vw
ν(C-H)	-	3004 vw	3018 vw	3020 vw	3023 vw
δ(NH2)	1649 m	1650 vw	1641 s	1641 s, br	1632 m
ν(C-C), γ(C-C-C)	1585 s	1611 w	1622 s	1609 m	1612 m
ν(C-C)	-	-	1563 s	1562 m	-
ν(C=N)	1494 m	1508 vw	1526 s	1520 s	-
ν(C-C)	1429 w	1436 vw	1466 m	1455 m	1459 vw
ν(C-NH2), ν(C-C)	1329 w	1331 w	1366 m	1346 m	1345 w
ν(C-N)	1267 m	1271 w	1295 m	1283 m	1278 w
γ (C-H)	1204 m	1212 w	1218 m	1213 s	1213 w
γ (C-H), γ(C-C-C)	1055 w	1053 s	1059 m	1059 m	1059 m
γ(C-C-C), ring breathing	976 s	990 s	1026 s	1022 s	1019 w
ω(NH2), τ(NH2)	838 sh	845 m	858 m	853 m	853 s
β(C-H)	813 vs	-	829 s	828 s	-
γ(C-C-C), ring deformation	664 s	666 w	678 w	669 vw	663 m
γ(C-N-C), ω(ring), ρ(NH2)	524 s	535 w	527 s	531 s	533 vw
γ(C-NH2)	442 w	437 m	-	-	-
β(C-C-C)	-	408 w	-	417 m	403 m
τ(NH2)	-	230 w	-	-	243 vw
Lattice vibration	-	117 s	-	-	114 s

νs: tretching; δ: bending; γ: in plane bending; β: out of plane bending; ω: wagging; τ: torsion; ρ: rocking; s: strong; m: medium; w: weak; vw: very weak; sh: shoulder; br: broad. ataken from [16].

The bending vibration of the NH_2_ group in the free 4AP gives a vibration peak at the wavenumber of 1649 and 1650 cm^–1^ in the FT-IR and FT-Raman spectra, respectively. This vibration peak occurred at the wavenumber of 1641 cm^–1^ in the FT-IR spectra of both complexes, while, occurred at a wavenumber of 1632 cm^–1 ^in the FT-Raman spectrum of complex **2** . Due to forming new complexes of the 4AP, this vibration mode has shifted to the lower wavenumber region of 8 cm^–1^ in the FT-IR spectra of complexes **1** and **2** . The same vibration mode has shifted to the lower wavenumber region of 18 cm^–1^ in the FT-Raman spectrum of complex **2** .

However, it is quite difficult to fully explain the 1680–1600 cm^–1^ wavenumber region of the vibration spectra of substances containing water molecules that are free or bonded with the NH_2_ group. Because, in the vibration spectrum of a complex that contains both NH_2_ group and water molecules in its structure, the bending vibrations of both the NH_2_ group and the water molecule occur in this wavenumber region. In addition, these vibrationmodes overlap because they have almost the same wavenumber. Some other researchers have also addressed this situation in their studies [14,40].

The ring breathing vibration mode of the free 4AP yields a vibration peak at 976 and 990 cm^–1^ wavenumbers in its FT-IR and FT-Raman spectra, respectively, while this vibration peak occurs at 1022 and 1019 cm^–1^ wavenumbers in the FT-IR and FT-Raman spectra of complex **2** , respectively. This vibration peak occurred in the FT-IR spectrum of complex **1** at 1026 cm^–1^ wavenumber. Due to the 4AP forming new complexes, this vibration mode has shifted to the higher wavenumber region of 50 and 46 cm^–1^ in the FT-IR spectra of complexes **1** and **2** , respectively. The same vibration mode occurred at 1019 cm^–1^ wavenumber in the FT-Raman spectrum of complex **2** , and, which shifted to the higher wavenumber region of 43 cm^–1^ relative to its value in that free 4AP. This vibration mode is one of the vibration modes, which shows compound formation with TM atoms of ligand molecules such as pyridines and aminopyridines, which have ring structure, and is most affected by this compound formation [6,7,13,15,16,18].

The tentative assignments and wavenumbers of some important vibration bands of the 4AP in the complexes **1** and **2** spectra are given in Table 5 together with the wavenumbers of the free state 4AP. The wavenumbers of the vibration bands most affected by the compounding of the 4AP are given in bold form in Table 5.

In the vibration spectra of complexes **1** and **2** , the observed small frequency shifts of the 4AP are thought to be caused by the changes in environmental conditions of this ligand molecule in the complexes, as well as the interaction of its internal vibration modes with the vibration modes of the M–N band that make up the crystal structure [5–7,15,16,18,37].

#### 3.2.2. [Ni(CN)4]2- group vibrations

The vibration wavenumbers of the [Ni(CN)_4_]^2– ^ions in the FT-IR and FT-Raman spectra of complexes **1** and **2** are given together in Table 6 to compare them with the vibration wavenumber of the compound K_2_[Ni(CN)_4_]·H_2_O. Sharp bands [ν(CN) stretch vibration bands] occurring in the spectra of compounds containing cyanide groups in the wavenumber range of 2200–2000 cm^–1^ and varying in intensities from weak to very severe are the most characteristic bands of the cyanide groups [5,41].

**Table 6:** The vibrational wavenumbers (cm–1) Ni(CN)4ion in complexes 1 and 2. The bands in the Raman spectra appear within parentheses.

Assignmenta	K2[Ni(CN)4]·H2O	1	2
ν1(C≡N), A1g	(2160) vs	(-)	(-)
ν4(C≡N), B1g	(2137) m	(-)	(2132) vs
ν8(C≡N), Eu	2122 vs	2169 vs (bridge)2141 m (terminal)	2170 vs (bridge)2136 s (terminal)
ν9(Ni–CN), Eu	544 w	561 w	565 w
δ(Ni–CN), B2g	(488) w	(-)	(482) w
π(Ni–CN), A2u	442 w	449 sh	448 w
δ(Ni–CN), Eu	420 s	437 s	417 s
π(Ni–CN), Eg	(303) s	(-)	(-)

ν: stretching; δ: in plane bending; π: out-of-plane bending; vs: very strong; s: strong; m: medium; w: weak; vw: very weak. a taken from [42]

The free[Ni(CN)_4_]^2–^ is an ionic structure with 21 fundamental vibrations and D_4h_ symmetry because it consists of nine atoms. The structural and vibration properties of the [Ni(CN)_4_]^2–^ ion have been meticulously studied by some researchers in the past [42,43]. Some of the 21 vibration modes of the [Ni(CN)_4_]^2–^ ion are IR active, some Raman active, others both IR and Raman active, while some are neither IR nor Raman active [42,43]. When the CN ligands in the [Ni(CN)_4_]^2–^ ion are considered as point structures, it is calculated that the [Ni(CN)_4_]^2–^ ion will have nine vibration modes. The IR active of these nine vibration modes are *ν* (C≡N) E *_u_* , *ν* (Ni–CN) E *_u_* , *π* (Ni–CN) A_2_ *_u_* , and *δ* (Ni–CN) E *_u_* . Since all four vibration bands are observed in the IR spectra of the complexes **1** and **2** , the [Ni(CN)_4_]^2–^ ions in the complexes **1** and **2** have a square planar arrangement. Vibration mode assignments of Ni(CN)_4_ group vibrations in complexes were assigned according to the work of McCullough [42], who analyzed vibration data for [Ni(CN)_4_]^2– ^ion in Na_2_Ni(CN)_4_ compound. The vibration wavenumbers of the [Ni(CN)_4_]^2–^ ion in complexes **1** and **2** are given in Table 6.

It should be remembered that the Raman spectrum of complex **1** cannot be taken for technical reasons. Although ν(C≡N), A *_1g_* stretching vibration band in the Raman spectrum of the K_2_[Ni(CN)_4_]·H_2_O compound was observed in the 2160 cm^–1^ wavenumber this band was not observed in the Raman spectrum of complex **2** . In the Raman spectrum of the K_2_[Ni(CN)_4_]·H_2_O compound, ν(C≡N), B *_1g_* stretching vibration band has been observed at a wavenumber of 2137 cm^–1^, but for the complex **2** of this band, it has been observed as shifted to the low frequency range of 5 cm^−1^.

In the IR spectrum of the starting compound K_2_[Ni(CN)_4_]·H_2_O, stretching vibration band [ν(C≡N), E *_u_* ] was observed at 2122 cm^–1^ wavenumber. This vibration band showed an interesting property by dividing into two different vibration bands in the IR spectra of complexes **1** and **2** . These split vibration bands appeared in complex **1** at wavenumbers 2169 and 2141 cm^–1^, and in complex **2** at wavenumbers 2170 and 2136 cm^–1^.

 In order for the splitting event to occur in a vibration band, some of the functional group that constitutes that vibration band in the compound must have different properties. When Figures 1–4 showing the molecular structures of complexes **1** and **2** are examined, it is seen that two cyanide groups in trans state according to the Ni atom in the structure of Ni(CN)_4_ ions are bound to zinc TM atoms, while, the other two cyanide groups in trans state are not bound. The presences of cyanide groups with these two different properties have caused the cyanide vibration band to split in the FT-IR spectra of complexes **1** and **2** .

In the IR spectrum of a compound, the stretching vibrations of both the bridging functional groups and the nonbonding (terminal) functional groups usually shift to the high frequency region according to their free states. The high frequency shift values of the bridge functional group formed by the division of the cyanide stretching peak are 47 and 48 cm^–1^ for complexes **1** and **2** , respectively, while the high frequency shift values of the terminal functional group are 19 and 14 cm^–1^. As can be seen in our current study, the shifting value to the high frequency region is always higher amount in bridging functional groups than terminal functional groups. The reality of this situation can be seen from previous studies both by us and other researchers [6,17,37,38].

However, the values that the frequencies of cyanide stretching vibrations in the IR spectrum of a compound can take depend on the electronegativities, coordination numbers and oxidation states of the metals forming that compound [4–7,42,43].

The peak of the stretching vibration [ν_9_(Ni–CN), E *_u_* ] of the starting compound K_2_[Ni(CN)_4_]·H_2_O was observed in its IR spectrum in the wavenumber region of 544 cm^–1^, while this peak of vibration shifted to the high wavenumber region of about 17 cm^–1^ in the IR spectrum of complex **1** . The same vibration peak has shifted to 21 cm^–1^ high wavenumber region in the IR spectrum of complex **2** .

The peak of the in-plane bending vibration of the [δ(Ni–CN), B *_2g_* ] of the starting compound K_2_[Ni(CN)_4_]·H_2_O was observed in its Raman spectrum in the wavenumber region of 488 cm^–1^, while, this peak of vibration shifted to the low wavenumber region of about 6 cm^–1^ in the Raman spectrum of complex **2** . 

The peak of the out-of-plane bending vibration of the [π(Ni–CN), A *_2u_* ] of the starting compound K_2_[Ni(CN)_4_]·H_2_O was observed in its IR spectrum in the wavenumber region of 442 cm^–1^, while, this peak of vibration shifted to the high wavenumber region of about 7 cm^–1^ in the IR spectrum of complex **1** . The same vibration peak has shifted to 6 cm^–1^ high wavenumber region in the IR spectrum of complex **2** .

The in-plane bending vibration [δ(Ni–CN), E *_u_* ] peak occurred at 420 cm^−1^ wavenumber in the starting compound, while the same vibration peak shifted to the region of high wavenumber of 17 cm^−1^ in complex **1** . The same vibration peak shifted to the low wavenumber region of about 3 cm^−1^ in the IR spectrum of complex **2** .

The peak of the out-of-plane bending vibration of the [π(Ni–CN), E *_g_* ] of the starting compound K_2_[Ni(CN)_4_]·H_2_O was observed in its Raman spectrum in the wavenumber region of 303 cm^–1^, while, this vibration peak was not observed in the Raman spectrum of complex **2** .

In the vibration spectra of the complexes examined here, it is expected that some new stretching vibration bands will appear at certain wavenumbers, which location varies depending on the electron donor atoms (N, S and O …) of the ligand molecule [5, 44,45]. The new M-N stretching vibrations resulting from the formation of these crystalline compounds were obtained in their IR spectrum as (468 w, 368 s and 292 s) cm^–1 ^and (480 vw, 355 m and 295 m) cm^–1^ for complexes **1** and **2** , respectively. Situations similar to these results have been observed in the studies of other researchers [44,45].

As a result of the spectroscopic examination of the complexes previously synthesized as powder with 4AP by other researchers and the complexes obtained as crystal by us, it was observed that similar changes occurred in almost the same vibration peaks [9,15,16,18].

#### 3.2.3. The vibrations of H2O guest water molecule

The water molecule has an angle of approximately 104.52° between the hydrogen bonds that form it, the oxygen atom in its structure is negatively charged and the hydrogen atoms are positively charged, so the water molecule is a polar molecule [46–49]. For this reason, the water molecule is an IR active molecule. Usually the water molecule is a weak Raman scattering substance, so its Raman spectrum is very weak. The water molecule has theoretically calculated asymmetric stretching at 3625 cm^–1^ wavenumber, symmetrical stretching at 3520 cm^–1^ wavenumber and O-H bending vibrations at 1641 cm^–^^1^ wavenumber. These theoretical vibration modes experimentally were obtained in the IR spectroscopy at 3431 and 1641 cm^–1^ wavenumbers and in the Raman spectroscopy, as two vibration peaks at 3448 and 1648 cm^–1^ wavenumbers in our laboratory studies. Due to the overlap of asymmetric and symmetric stretching vibrations of the water molecule by interacting with each other, a very wide vibration band with a peak at 3351 cm^–1^ wavenumbers formed in its vibration spectrum, respectively. Similar situations have been observed in previous studies by other researchers [46,47].

In the literature, a huge amount of different scientific studies can be found in which water molecules are used both as ligand molecules and as guest molecules [49–52]. If there are some bound water molecules in the structure of a synthesized compound, its stretching vibration band is generally observed as a very broad band between 3500 and 3000 cm^–1^ wavenumber. If there are some unbound water molecules in the structure of a synthesized compound, its stretching vibration band is generally observed as a very sharp band between 3550 and 3650 cm^–1^ wavenumbers [5,37,51].

Examining the IR spectra of complexes **1** and **2** reveals some interesting and important situations. When examining the crystal structures of the complex **1** in which N-H···O and O-H···N bonds made by water molecules are effective in the formation of the 3D structure of complex **1** , it was given. The spectroscopic evidence of this is that the symmetrical stretch vibration of the NH_2_ group in the IR spectrum of complex **1** is wider than that of complex **2** . This is because of is coincide the O-H stretching vibrations of the bonded some water molecules and the symmetrical stretching vibration of the NH_2_ group. At the same time, a sharp peak at 3607 cm^–1^ in the IR spectrum of complex **1** proves the presence of some unbounded water molecules.

The same sharp peak was seen as a very weak band at 3601 cm^–1^ in both the IR and Raman spectra of complex **2** . In other words, these water molecules in the complexes can be found both as a member of the host structure and as guest molecules. It is also seen that the intensities of these sharp peaks in the IR spectra of the complexes are directly proportional to the number of guest water molecules in the complex. 

In the IR spectra of complexes **1** and **2** , it is seen that the guest water molecules in their structures have OH bending vibrations at wavenumbers of 1622 and 1621 cm^–1^, respectively. In the Raman spectrum of complex **2** , it is seen that the guest water molecules in its structure has OH bending vibration at wavenumber of 1612 cm^–1^ as a weak intensity band.

### 3.3. Thermal behavior of the complexes 1 and 2

**Figure 7:**
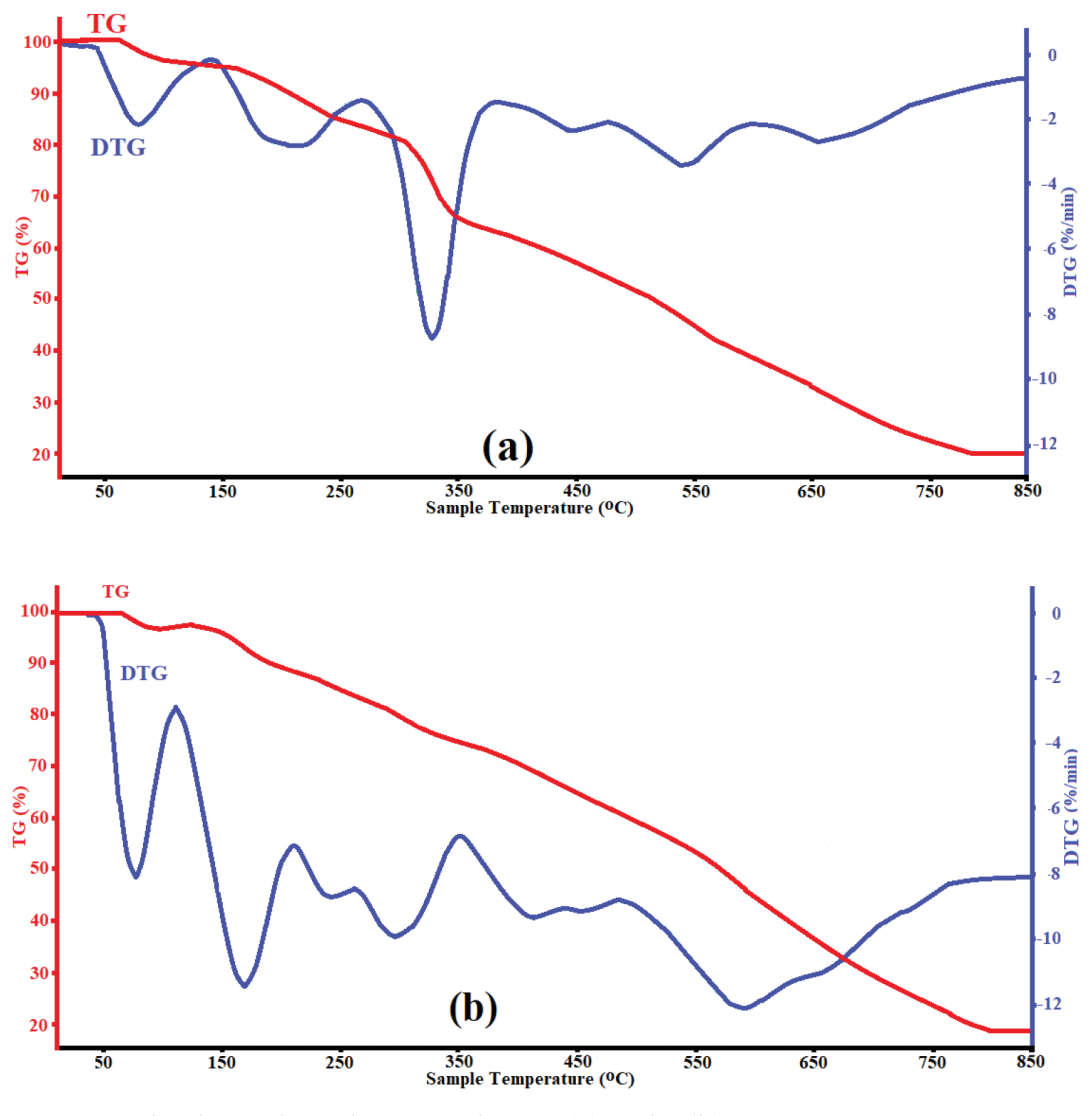
The thermal graphics complexes 1 (a) and 2 (b).

The thermal behaviors of complexes **1** and **2** , corresponding to the increase in the ambient temperature, were investigated in the nitrogen atmosphere and in the temperature range (25–850) ℃. Thermal behavior graphics (TGA and DTG) belonging to complexes **1** and **2** are given in Figures 7a and 7b. A five-stage thermal behavior was observed in both complexes. The thermal behavior graphs of complexes **1** and **2** are shown in Figure 7.

However, each of the five steps seen in the thermal behavior of complexes is not precisely separated from the others. Experimental evidence for this is the absence of plateaus at certain temperature ranges in the thermal behavior graphs of both complexes. That is, while a thermal step continues, the next thermal step begins to occur. As can be seen from Figures 7a and 7b, both complexes are thermally stable from the initial temperature of 25 ℃ to temperatures of 67 and 73 ℃, respectively. However, by heating the complexes, both complexes gradually lose their guest water molecules first.

The six molecules of guest water in complex **1** are separated from its structure in the first step of thermal decomposition in the temperature range of 67 to 255°C with two DTG_max_ of 84 and 205 °C. The amount of guest water molecules separated from the structure of complex **1** is theoretical 20.61% and experimental 19.31%, respectively.

The separation of the NH_2_ groups of the two ligand molecules in complex **1** from its structure occurs in the second step in the temperature range of 255 to 378 °C with a DTG_max_ of 342 °C. The amount of NH_2_ groups separated from the structure of complex **1** is 6.11% theoretical, 6.10% experimental, respectively.

In the third step of the thermal decomposition of complex **1** , the remaining pyridine rings of the two 4AP ligand molecules are separated from its structure in the temperature range of 378 to 472 °C with a DTG_max_ of 448 °C. The amount of pyridine rings separated from the structure of complex **1** is 29.79% theoretical and 28.61% experimental, respectively.

In the fourth step of the thermal decomposition of complex **1** , the cyanide parts of the Ni(CN)_4_ groups are separated from its structure in the temperature range of 472 to 611 °C with a DTG_max_ of 551 °C. The amount of cyanide parts separated from the structure of complex **1** is 19.84% theoretical and 21.57% experimental, respectively.

In the fifth and last step of the thermal decomposition of complex **1** , the zinc and nickel atoms are completely separated and purified from other organic parts in the temperature range between 611and 792 °C with a DTG_max_ of 663 °C. The remaining amount of zinc and nickel atoms in the experimental environment is 23.66% theoretical and 24.31% experimental, respectively.

The steps for the thermal degradation of complex **2** are also similar to the thermal degradation steps of complex **1** . The one molecule of guest water in complex **2** is separated from its structure in the first step of thermal decomposition in the temperature range of 73 to 126 °C with a DTG_max_ of 96 °C. The amount of guest water molecule separated from the structure of complex **2** is theoretical 2.90% and experimental 3.21%, respectively.

The separation of the NH_2_ groups of the four ligand molecules in complex **2** from its structure occurs in the second step in the temperature range of 126 to 192 °C with a DTG_max_ of 164 °C. The amount of NH_2_ groups separated from the structure of complex **2** is 10.32% theoretical, 9.83% experimental, respectively.

In the third step of the thermal decomposition of complex **2** , the remaining pyridine rings of the four 4AP are separated from its structure in the temperature range of 192 to 361 °C with two DTG_max_ of 240 and 293 °C. The amount of pyridine rings separated from the structure of complex **2** is 50.32% theoretical and 48.92% experimental, respectively.

In the fourth step of the thermal decomposition of complex **2** , the cyanide parts of the Ni(CN)_4_ groups are separated from its structure in the temperature range of 361 to 493 °C with a DTG_max_ of 412 °C. The amount of cyanide parts separated from the structure of complex **2** is 16.76% theoretical and 17.23% experimental, respectively.

In the fifth and last step of the thermal decomposition of complex **2** , the zinc and nickel atoms are completely separated and purified from other organic parts in the temperature range between 493 and 790 °C with a DTG_max_ of 588 °C. The remaining amount of zinc and nickel atoms in the experimental environment is 19.69% theoretical and 20.78% experimental, respectively.

Similar thermal decomposition steps have been observed for other Hofmann-type complexes and Hofmann-type clathrates [6,7,37,38].

### 3.4. Magnetic moments of the complexes 1 and 2

Since the Ni(CN)_4_ group in complex **1** has a square planar structure, the nickel atom in this group does not have a magnetic moment. At the same time, the zinc atom, which has a distorted tetrahedral geometry in this complex, also has a d^10^ closed shell electron arrangement, so its unpaired electron number is zero. For these reasons, the theoretically calculated magnetic moment value of complex **1** is 0.000 BM.

Since the Ni(CN)_4_ group has a square planar structure in complex **2** , the nickel atom in this group also does not have a magnetic moment. At the same time, the copper atom with distorted octahedral geometry in this complex has the d^9^ electron arrangement, so its unpaired electron number is one. For these reasons, the theoretically calculated magnetic moment value of complex **2** is found to be 1.732 BM.

Experimental magnetic moment values of complexes measured by Gouy method under laboratory conditions are 0.004 BM for complex **1** and 1.692 BM for complex **2** . As can be seen from here, the experimentally measured magnetic moment values of both complexes **1** and **2** are quite compatible with the theoretically calculated magnetic moment values. 

## 4. Conclusion

In our study, structural properties of [Zn(II)(4AP)_2_Ni(µ-CN)_2_(CN)_2_]·6H_2_O and [Cu(II)(4AP)_4_Ni(µ-CN)_2_(CN)_2_]·H_2_O clathrates obtained by chemical methods were analyzed and characterized by SC-XRD, FT-IR and FT-Raman spectroscopy, thermal and elemental analysis methods. Comparing the FT-IR and FT-Raman spectral data of these clathrates with their crystallographic data shows that the results are quite consistent. The clathrates crystallized in orthorhombic and monoclinic crystal systems and in *Cmcm* and *C2c* space groups, respectively. In the formation of crystal structures of complexes **1** and **2** , the 4AP has contributed by binding to the TM atom from its ring nitrogen atom and from the hydrogen atoms of the NH_2_ group to the nitrogen atoms of the cyanide group. The guest water molecules also contributed to the formation of the crystal structures of complexes **1** and **2** by making N-H···O and O-H···N hydrogen bonds too. The metal-nitrogen, hydrogen-nitrogen and hydrogen-oxygen bonds play the biggest role in the formation of the 3D crystal structure of complex **1** , and metal-nitrogen and N-H···N bonds play the biggest role in the formation of the 2D crystal structure of complex **2** .

The Zn(II) ion in the structure of complex **1** is located at the center of inversion and coordinates with two nitrogen atoms from the cyanide ligands and two nitrogen atoms from the 4AP, thus showing a distorted tetrahedral coordination geometry. Likewise, the Ni(II) ion in the structure of complex **1** is also coordinated by four carbon atoms from the cyanide ligands, thus showing a square planar coordination geometry. The Cu(II) ion in the structure of complex **2** is located at the center of inversion and coordinates with two nitrogen atoms from the cyanide ligands and four nitrogen atoms from the 4AP, thus showing a distorted octahedral coordination geometry. Likewise, the Ni(II) ion in the structure of complex **2** is also coordinated by four carbon atoms from the cyanide ligands, thus showing a square planar coordination geometry.

All structural properties of the complexes **1** and **2** and their spectral data showed that they have Hofmann-type-like 4AP water clathrates and polymeric structures with two TM atoms. In some future studies, it can be investigated how the crystal structures of Hofmann-type compounds or clathrates are bound to other TM atoms using the same 4AP and guest water molecule.

## Supplementary material

Crystallographic data for the structural analysis has been deposited with the Cambridge Crystallographic Data Centre, CCDC No. 1968719 for **1** and 1565193 for **2** . Copies of this information may be obtained free of charge from the Director, CCDC, 12 Union Road, Cambridge CB2 1EZ, UK (fax: +44-1223-336033; e-mail: deposit@ccdc.cam.ac.uk or www: http://www.ccdc.cam.ac.uk).
